# Galectin-3 Inhibition by a Small-Molecule Inhibitor Reduces Both Pathological Corneal Neovascularization and Fibrosis

**DOI:** 10.1167/iovs.16-20009

**Published:** 2017-01

**Authors:** Wei-Sheng Chen*, Zhiyi Cao, Hakon Leffler, Ulf J. Nilsson, Noorjahan Panjwani

**Affiliations:** 1 Program in Cell, Molecular and Developmental Biology, Sackler School of Graduate Biomedical Sciences, Tufts University, Boston, Massachusetts, United States; 2 New England Eye Center/Department of Ophthalmology, Tufts University, Boston, Massachusetts, United States; 3 Section of Microbiology Immunology and Glycobiology, Department of Laboratory Medicine, Lund University, Lund, Sweden; 4 Centre for Analysis and Synthesis, Department of Chemistry, Lund University, Lund, Sweden

**Keywords:** galectin, 33DFTG, angiogenesis, fibrosis, cornea

## Abstract

**Purpose:**

Corneal neovascularization and scarring commonly lead to significant vision loss. This study was designed to determine whether a small-molecule inhibitor of galectin-3 can inhibit both corneal angiogenesis and fibrosis in experimental mouse models.

**Methods:**

Animal models of silver nitrate cautery and alkaline burn were used to induce mouse corneal angiogenesis and fibrosis, respectively. Corneas were treated with the galectin-3 inhibitor, 33DFTG, or vehicle alone and were processed for whole-mount immunofluorescence staining and Western blot analysis to quantify the density of blood vessels and markers of fibrosis. In addition, human umbilical vein endothelial cells (HUVECs) and primary human corneal fibroblasts were used to analyze the role of galectin-3 in the process of angiogenesis and fibrosis in vitro.

**Results:**

Robust angiogenesis was observed in silver nitrate–cauterized corneas on day 5 post injury, and markedly increased corneal opacification was demonstrated in alkaline burn–injured corneas on days 7 and 14 post injury. Treatment with the inhibitor substantially reduced corneal angiogenesis and opacification with a concomitant decrease in α-smooth muscle actin (α-SMA) expression and distribution. In vitro studies revealed that 33DFTG inhibited VEGF-A–induced HUVEC migration and sprouting without cytotoxic effects. The addition of exogenous galectin-3 to corneal fibroblasts in culture induced the expression of fibrosis-related proteins, including α-SMA and connective tissue growth factor.

**Conclusions:**

Our data provide proof of concept that targeting galectin-3 by the novel, small-molecule inhibitor, 33DFTG, ameliorates pathological corneal angiogenesis as well as fibrosis. These findings suggest a potential new therapeutic strategy for treating ocular disorders related to pathological angiogenesis and fibrosis.

Corneal neovascularization is a vision-threatening condition affecting ∼1.4 million individuals each year in the United States alone.^1,2^ It is associated with a wide range of ocular disorders including infections, trauma, and inflammatory disorders of the ocular surface. The complications of corneal neovascularization include corneal scarring, edema, lipid deposition, and increased risk of graft rejection.^2–4^ Thus, there is tremendous interest in the development of effective strategies to prevent the growth of blood vessels in the cornea, not only to prevent corneal graft rejection, but also to treat numerous other inflammatory disorders of the ocular surface. Vascular endothelial growth factor (VEGF or VEGF-A) is one of the major proangiogenic factors of both physiological and pathological angiogenesis, and is consistently upregulated during diverse forms of pathological angiogenesis.^5,6^ The VEGF/VEGF receptor-2 (VEGFR-2)–targeting therapies, including neutralizing antibodies (bevacizumab and ranibizumab) and kinase inhibitors (sorafenib and sunitinib), have shown clinical benefits for cancer patients either as single agent or when combined with chemotherapy (reviewed in Ref. 7). In addition, the introduction of VEGF-targeting therapies in ophthalmology has revolutionized the treatment of wet age-related macular degeneration, diabetic macular edema, and retinal vein occlusion (reviewed in Ref. 8). There are also several clinical trials using anti-VEGF therapies to treat corneal neovascularization-related diseases including corneal graft rejection and pterygium (reviewed in Ref. 9). Although VEGF-targeting therapies are beneficial, resistance and nonresponsiveness to the VEGF-targeting therapies are still a major challenge in the clinic.^10,11^ In fact, up to 45% of patients with choroidal neovascularization secondary to age-related macular degeneration do not respond to bevacizumab treatment.^12,13^ For assessment of long-term usage of anti-VEGF therapy in clinics, the Seven Year Update of Macular Degeneration Patients (SEVEN-UP) study showed that one-third of patients treated with an anti-VEGF therapy had worsened vision and that 98% of study eyes had macular atrophy.^14,15^ Clearly, it is important to develop more rational anti-VEGF/VEGFR-2 regimens and to target other key players in the process of angiogenesis.

Pathological fibrosis, or excessive tissue scarring, accounts for as many as 45% of deaths in the United States.^16^ Fibrosis is characterized by excessive extracellular matrix (ECM) accumulation that impairs normal functions of tissues/organs. Ocular tissues are also susceptible to fibrotic disorders, which may occur in cornea, conjunctiva, trabecular meshwork, lens, and retina (reviewed in Ref. 17). Corneal scarring, a leading cause of global blindness, is often caused by injury, trauma, and/or infection to the eye; approximately 4.9 million are totally blind from trachomatous corneal scarring.^18^ Current treatment options to control corneal scarring are limited and their outcomes are typically poor. There currently are no Food and Drug Administration–approved drugs that selectively reduce scar formation. Corneal wound healing is an intricate process, and the factors that govern the development of scar tissue after ocular injury and pathological neovascularization are not well understood. It is known, however, that the fibrogenic process is stimulated by inflammatory cell–derived cytokines, especially transforming growth factor-β (TGF-β), which promotes activation of myofibroblasts leading to increased ECM deposition, scarring, and dysfunction of the tissue with substantial vision loss. As uncontrolled and persistent myofibroblast activation leads to pathological fibrosis, it is widely believed that reducing the number and activity of myofibroblasts is crucial to complete the healing process and to restore the normal tissue functions.^19^

Galectins are a family of animal lectins that bind β-galactosides.^20,21^ Our recent studies demonstrated that corneal stromal expression of galectin-3 is markedly upregulated in sterile and nonsterile inflammation.^22,23^ Mechanistically, galectin-3 is an important modulator for VEGF/VEGFR-2 signaling pathway. Galectin-3 is upregulated in response to hypoxia, and previous studies in our laboratory demonstrated that galectin-3 modulates the functions of VEGFR-2 in endothelial cells and that galectin-3 deficiency attenuates inflammatory angiogenesis in vivo.^24–27^ In addition, we have shown that galectin-3 modulates not only VEGF- but also basic fibroblast growth factor (bFGF)-mediated angiogenesis in vivo.^28^ Considering that galectin-3 is a key molecule that modulates the process of pathological angiogenesis and that galectin-3 knockout mice have no defects in developmental angiogenesis, targeting galectin-3 makes it an attractive strategy to treat pathological angiogenesis-related diseases.

Independent of its proangiogenic function, galectin-3 also promotes fibrosis.^29^ Nonocular studies using preclinical models of lung, kidney, heart, and liver fibrosis^29–32^ have shown that galectin-3–deficient mice are resistant to myofibroblast activation and procollagen deposition and are thus protected from fibrosis. Subsequent mechanistic studies have shown that galectin-3 is required for TGF-β–mediated myofibroblast activation and matrix production^31^ and that galectin-3 promotes an M2 macrophage state in mice, which is associated with increased fibrosis.^33^ To the best of our knowledge, galectin-3 has thus far not been investigated with respect to its profibrotic role in the eye. In the current study, we targeted galectin-3 to control both corneal neovascularization as well as fibrosis in mouse animal models.

## Methods

### Animals

C57BL/6 mice (male, 8–10 weeks old) were obtained from Jackson Laboratory (Bar Harbor, ME, USA). Previous studies have demonstrated that male mice display stronger angiogenic response^34,35^; therefore, male mice were chosen in this study. All experimental procedures were conducted in compliance with the ARVO Statement for the Use of Animals in Ophthalmic and Vision Research and were approved by the Institutional Animal Care and Use Committee at Tufts University.

### Cell Culture

Human umbilical vein endothelial cells (HUVECs; Lonza, Walkersville, MD, USA) were maintained in EBM-2 medium supplemented with EGM BulletKit (Lonza), penicillin (100 units/mL), and streptomycin (100 μg/mL; Gibco, Grand Island, NY, USA). Cells were seeded on plates and dishes coated with 0.1% gelatin (Sigma-Aldrich Corp., St. Louis, MO, USA), and were used between passages 3 and 5. Human corneal fibroblasts were isolated from donor corneal rims as described before.^36^ The primary corneal fibroblasts were maintained in MEM medium (Invitrogen, Carlsbad, CA, USA) supplemented with 10% FBS and were used between passages 3 and 8.

### Galectin-3 Inhibitor

A small molecular weight inhibitor of galectin-3, 3,3′-dideoxy-3,3′-bis-[4-(3-fluorophenyl)-1H-1,2,3-triazol-1-yl]-1,1′- sulfanediyl-di-β-D-galactopyranoside (33DFTG, previously called TD139),^31,37,38^ was used in this study. The inhibitor competes with galectin-3′s canonical carbohydrate binding site with a K_d_ of approximately 14 nM.^31^ As a rule of thumb, concentrations used for our in vitro studies were ∼100-fold higher than the K_d_ of the inhibitor, and dose response of 33DFTG in each assay was determined separately. The maximum inhibitory effect of 33DFTG varied in different assays; 0.1 μM 33DFTG completely inhibited VEGF-A−induced cell migration in the chemotaxis assay, whereas 5 μM 33DFTG inhibited ∼50% of VEGF-A−induced sprouting in the three-dimensional (3D) sprouting assay.

### Preparation and Instillation of Eye Drop Formulation of 33DFTG

To make 0.01% (wt/vol or 154.2 μM) 33DFTG in 1.65% (wt/vol) hydroxyethyl cellulose (HEC; EMD Millipore, Billerica, MA, USA), 2 mg 33DFTG was added to calcium- and magnesium-free PBS (pH 7.4; Invitrogen) and mixed with 330 mg HEC with an agitator for 2 hours at 25°C, and the volume was adjusted to 20 mL. Lower doses of 33DFTG eye drops were prepared by dilution with unloaded 1.65% HEC. After corneal injuries, eye drops were instilled twice a day (10 μL per eye) until the end of the experiments.

### Animal Models of Corneal Neovascularization and Fibrosis

Distinct mouse animal models of corneal neovascularization and fibrosis were used. To induce corneal neovascularization, corneas were subjected to a less severe form of corneal injury involving silver nitrate cautery for 5 seconds that damages central cornea with little damage to the peripheral cornea. This technique produces robust neovascularization by day 5 post treatment that is almost completely cleared by 2 weeks. To induce more severe corneal injury and fibrosis, we employed another well-established mouse model of chemical injury, in which corneas were subjected to alkali injury and the entire corneal epithelium, from limbus to limbus, was removed. With this technique, corneal opacity and fibrosis peaks at 1 to 2 weeks and persists at least 1 month. Details of both methods are described below.

#### Mouse Model of Corneal Neovascularization.

Mice were anesthetized with intraperitoneal injection of ketamine (90–120 mg/kg) and xylazine (10 mg/kg), and a silver nitrate applicator coated with 75% silver nitrate and 25% potassium nitrate (Grafco, Atlanta, GA, USA) was applied on the surface of the central cornea of the right eye of each animal for 5 seconds under a surgical microscope.^22^ The corneas were rinsed with PBS, and ophthalmic antibiotic ointment (Alcon, Fort Worth, TX, USA) was topically applied to the operated eyes to prevent infection. To assess the antiangiogenic effect of the inhibitor, 10 μL 33DFTG (325 ng in 0.5% DMSO/PBS, equal to 50 μM) or vehicle (0.5% dimethyl sulfoxide [DMSO]/PBS) was administered by subconjunctival injections on postsurgery days 0, 2, and 4 using a 32-gauge needle with a 10-μL syringe (Hamilton, Reno, NV, USA). For subconjunctival injections, the concentration of 33DFTG used was 10-fold higher than the concentration exhibiting maximum inhibition in the 3D sprouting assay. This concentration is similar to that used in a nonocular study.^31^ On day 5 post surgery, corneas were harvested and processed for whole-mount staining with anti-CD31 antibody to reveal blood vessels in the mouse cornea.

#### Mouse Model of Corneal Fibrosis.

Corneal fibrosis was induced in the mouse model as previously described.^39^ Mice were anesthetized; sodium hydroxide (1.5 μL 0.15 N) was applied to the central cornea of the right eye of each animal for 1.5 minutes and was immediately rinsed away with PBS.^39^ The corneal and limbal epithelium was removed by an Algerbrush (Ambler Surgical, Exton, PA, USA). Ophthalmic antibiotic ointment was topically applied to the operated eyes. Corneal opacification was scored on days 7 and 14 post surgery by slit-lamp biomicroscopy (SL-D7; Topcon, Tokyo, Japan). A scoring system^40^ ranging from 0 to 4+ was used (0, clear; 1+, opacity area less than 50% and pupil clearly visible; 2+, opacity area more than 50% and pupil visible; 3+, pupil invisible but iris visible; 4+, both pupil and iris invisible). On day 14 post surgery, corneas were harvested and processed for Western blotting and immunofluorescence staining with anti-α-smooth muscle actin (α-SMA) antibody to reveal fibrosis. To assess the antifibrotic effect of the inhibitor, 10 μL 33DFTG (325 ng in 0.5% DMSO/PBS, equal to 50 μM) or vehicle (0.5% DMSO/PBS) was administered by subconjunctival injections on alternate days from day 1 until day 13 post surgery using a 32-gauge needle with a 10-μL syringe.

### Whole-Mount Immunofluorescence Staining of Corneas to Visualize Blood Vessels

The enucleated eyes were fixed with 4% paraformaldehyde (Electron Microscopy Science, Hatfield, PA, USA) in PBS for 30 minutes at 4°C, and then the corneas were excised, washed with PBS, and fixed again with iced methanol (15 minutes, 25°C) followed by three washes with 0.3% Triton X-100/PBS. Nonspecific binding sites were blocked with 10% donkey serum in 0.3% Triton X-100/PBS (blocking buffer), and the corneas were sequentially incubated in anti-mouse CD31 antibody (clone MEC 13.3, 1:100 dilution, overnight, 4°C; BD Pharmingen, San Diego, CA, USA) in blocking buffer, and Alexa Fluor 488 donkey anti-rat IgG in blocking buffer (1:300 dilution, 1.5 hours, 25°C; Invitrogen). After four washes with 0.3% Triton X-100/PBS, corneal flat mounts were prepared on glass slides using VECTASHIELD mounting medium (Vector Laboratories, Burlingame, CA, USA). Images were acquired by EVOS FL cell imaging system (Invitrogen). The CD31^+^ blood vessel area of each corneal flat mount was quantified by ImageJ software (http://imagej.nih.gov/ij/; provided in the public domain by the National Institutes of Health, Bethesda, MD, USA), and results were calculated as a percentage of the total corneal area outlined by the border of the outermost vessel of the limbal arcade.

### Immunofluorescence Staining to Visualize α-SMA

Frozen sections (8 μm) of the mouse eyes were fixed with iced acetone (10 minutes, 25°C), blocked with Image-iT FX signal enhancer (30 minutes, 25°C; Invitrogen), and immunostained using rabbit anti-α-SMA (NB600-531, 1:200 dilution in 5% BSA/PBS, overnight, 4°C; Novus Biologicals, Littleton, CO, USA) and Alexa Fluor 488–conjugated anti-rabbit IgG secondary antibody. The tissue sections were mounted with ProLong gold antifade mounting medium with 4′,6-diamidino-2-phenylindole (DAPI; Invitrogen). Fluorescence images were acquired by Leica TCS SPE imaging system (Leica Microsystems, Inc., Buffalo Grove, IL, USA). Negative controls with isotype primary antibody were also included. No signal was detected in the negative controls (data not shown).

### Western Blot Analysis

Western blotting was performed as described before.^22^ Briefly, protein extracts of mouse corneas were prepared in a radioimmunoprecipitation (RIPA) buffer supplemented with 1% SDS and Halt protease and phosphatase inhibitor cocktail (Thermo Fisher Scientific, Waltham, MA, USA). Four corneas were pooled and considered one biological replica. Protein concentration was determined by Bradford-based protein assay (Bio-Rad protein assay). Equal amounts of lysates (30 μg protein) were subjected to electrophoresis in 4% to 15% gradient SDS-PAGE gels (Bio-Rad, Hercules, CA, USA). Protein blots of the gels were blocked with Odyssey blocking buffer (OBB; Li-Cor Biosciences, Lincoln, NE, USA) and incubated overnight with primary antibodies: rabbit anti-α-SMA (ab5694, 1:2000 dilution in OBB; Abcam, Cambridge, MA, USA), goat anti-connective tissue growth factor (CTGF) (clone L-20, 1:500 dilution in OBB; Santa Cruz Biotechnology, Dallas, TX, USA), and mouse anti-β-actin (clone AC-15, 1:10,000 dilution in OBB; Santa Cruz Biotechnology). The secondary antibody used was anti-rabbit IgG IRDye 800CW, anti-goat IRDye 800CW, and anti-mouse IgG IRDye 680LT (1:10,0000 dilution in OBB; Li-Cor Biosciences). Blots were then scanned with the Odyssey Infrared Imaging System, and relative band intensity was quantified by Image Studio v2.0 software (Li-Cor Biosciences).

### HUVEC Sprouting Assay

Human UVEC spheroids were generated by seeding primary HUVEC at passage 3 to 5 in each well of 384-well hanging-drop plates (3D Biomatrix, Ann Arbor, MI, USA) in complete EBM-2 medium containing 0.25% methyl cellulose (750 cells in 30 μL/well). After 18 hours, HUVEC spheroids were collected, resuspended in serum-free EBM-2 medium, and mixed with collagen solution (PureCol collagen; Advanced BioMatrix, San Diego, CA, USA; 2.2 mg/mL in M199 medium, pH adjusted to 7.4 with NaHCO_3_ and NaOH). To determine the effect of the inhibitor on VEGF-A–induced sprouting, aliquots of the HUVEC spheroid/collagen mixture (300 μL/well of 48-well plate) were pretreated with various concentrations of 33DFTG (0.01–10 μM) for 6 hours. After the 6-hour incubation period, 300 μL VEGF-A (100 ng/mL in serum-free EBM-2 medium; PeproTech, Rocky Hill, NJ, USA) was combined with 300 μL collagen mixture in the presence or absence of corresponding doses of 33DFTG. Plates were incubated for 24 hours at 37°C; cumulative sprout length of each sprout was calculated, and the results were expressed as fold change compared to VEGF-A–treated group. Because 33DFTG stock was dissolved in DMSO, all treatments contained 0.05% DMSO. Of note, sprout lengths vary from passage to passage (cells with higher passage number showed fewer sprouting length/numbers in response to the same concentration of VEGF-A).

### HUVEC Migration Assay

Human UVEC migration was performed using Transwell (6.5 mm) with 8-μm-pore polycarbonate membrane inserts (Corning Life Sciences, Tewksbury, MA, USA). The membranes were coated overnight with 0.1% gelatin (Sigma-Aldrich Corp.) at 37°C. Human UVECs were serum starved overnight with 1% FBS in M199 medium at 37°C, detached with StemPro Accutase (Thermo Fisher Scientific, Waltham, MA, USA) cell dissociation reagent, and resuspended in 1% FBS/M199 medium at 2 × 10^5^ cells/mL. An aliquot of resuspended cells (200 μL; 40,000 cells/well) was placed in the upper well of the Transwell inserts, and 1% FBS/M199 medium (600 μL) with or without VEGF-A (100 ng/mL) was mixed with varying concentrations of 33DFTG (0.01–1 μM) and placed in the lower well. After 3-hour incubation at 37°C, inserts were fixed in absolute methanol (6 minutes, 25°C) and stained with Giemsa stain (40 minutes, 25°C; Sigma-Aldrich Corp.) per manufacturer's instructions. Membranes were wiped free of cells on the upper surface and mounted with Permount mounting medium (Fisher Scientific, Waltham, MA, USA) on glass slides. The number of migrating cells per condition was counted in four random fields at 10× magnification and averaged, and the results were compared with the vehicle-treated control cells to calculate fold change in migration activity.

### HUVEC Cytotoxicity Assay

To assess the cytotoxic effect of the inhibitor, two reagents, calcein acetoxymethyl ester (calcein AM; Invitrogen) and WST-1 (Roche Life Science, Indianapolis, IN, USA), were used in this study. Human UVECs (5000 cells in 100 μL/well) were grown overnight at 37°C in a 96-well gelatin-coated plate. The cells were serum starved in 1% FBS/M199 medium and treated with 0.05% DMSO (vehicle control), 0.1% Triton X-100 (positive control), or 5 μM 33DFTG overnight at 37°C (three replicas/condition). For calcein AM–based assay, cells were incubated with 1 μM calcein AM in serum-free M199 medium, 30 minutes, 37°C. Absorbance was detected from the bottom using the FilterMax F5 multimode microplate reader (Molecular Devices, Sunnyvale, CA) at excitation/emission wavelengths of 485 nm/535 nm. For WST-1–based assay, 10 μL ready-to-use WST-1 reagent was added to each well and incubated for 3 hours at 37°C, and absorbance at 450 nm was measured using the FilterMax F5 multimode microplate reader.

### Flow Cytometry Analysis

To determine galectin-3 expression at the cell surface of primary human corneal fibroblasts and epithelial cells, the cells were lifted with a cell detachment solution (AccuMax; eBioscience, San Diego, CA, USA), washed with iced PBS, and then stained using Alexa Fluor 488–conjugated rat anti-galectin-3 antibody (clone M3/38; BioLegend, San Diego, CA, USA) or isotype control antibody (BioLegend) (both 1:100 dilution in the commercial cell staining buffer, 4°C, 45 minutes, BioLegend). The stained cells were fixed with 2% paraformaldehyde/PBS. Flow cytometry was performed on the BD FACSCalibur, and data were analyzed with the FlowJo software (version 9.5.2; Ashland, OR, USA). To determine intracellular galectin-3 expression, the detached cells were fixed with 4% paraformaldehyde/PBS (4°C, 10 minutes), washed with PBS, permeabilized in 1× BD Perm/Wash buffer (BD Biosciences, 4°C, 15 minutes), and then stained using anti-galectin-3 antibody or isotype control antibody as mentioned above except that the antibodies were diluted with 1× BD Perm/Wash buffer. The stained cells were fixed with 2% paraformaldehyde/PBS and subjected to flow cytometry.

### Treatment of Human Corneal Fibroblasts with TGF-β1 and Galectin-3

Recombinant galectin-3 was prepared as described before.^28,41^ Briefly, human galectin-3 cDNA was cloned into *Nco*I/*Hin*d III cut pKK233-2, transformed into BL21 Star (DE3)–competent cells, and plated onto LB agar containing ampicillin (50 μg/mL). The recombinant galectin-3 expression was induced by 0.1 mM β-D-1-thiogalactopyranoside (16 hours, 25°C) when optical density (OD)600 is 0.6. Bacterial pellets were resuspended in a lysis buffer (PBS supplemented with 2 mM EDTA and 4 mM β-mercaptoethanol). Cells were disrupted with a probe-type sonicator. The supernatant of the cell lysate was passed over a Lactosyl-Sepharose column (Sigma-Aldrich Corp.) and equilibrated with the lysis buffer. Bound proteins were eluted with 100 mM lactose in PBS. Lactose was then removed by dialysis against 5% glycerol in PBS at least four times. Endotoxin was removed by Detoxi-Gel endotoxin removing gel (Thermo Fisher Scientific), and endotoxin levels were detected by ToxinSensor chromogenic LAL endotoxin assay kit (Genscript, Piscataway, NJ, USA). Endotoxin levels of the recombinant galectin-3 were <0.1 EU/μg. To determine the profibrotic effect of galectin-3 in primary human corneal fibroblasts, the cells were serum starved overnight in 0.1% FBS/MEM medium, and then treated with varying concentrations of recombinant galectin-3 or TGF-β1 (0.5 ng/mL, PeproTech) for 24 hours in 0.1% FBS/MEM medium. Cell lysates were subjected to Western blotting using antibodies against α-SMA, CTGF, and β-actin as described above.

### Statistics

Data in all figures are presented as mean ± SEM. Data were analyzed using paired 2-tailed Student's *t*-test or 1-way ANOVA in Prism 6 (GraphPad SoftWare, Inc., La Jolla, CA, USA) as indicated in figure legends. *P* value of less than 0.05 was considered statistically significant.

## Results

### Galectin-3 Inhibition Reduces Corneal Angiogenesis In Vivo

**Figure 1:**
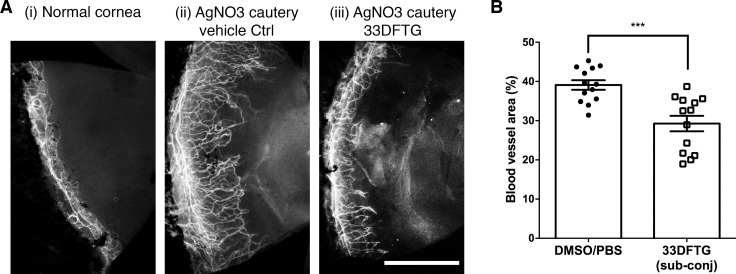
Galectin-3 inhibition by 33DFTG reduces corneal angiogenesis in vivo. Neovascularization was induced in mouse corneas by silver nitrate cautery as described in Methods. (**A**) Ten microliters of 33DFTG (325 ng) in PBS containing 0.5% DMSO or vehicle alone was administered by subconjunctival injections every other day. After 5 days, mice were killed, and flat mounts of corneas were stained with anti-CD31 to visualize blood vessels. Representative corneal flat mounts stained with anti-CD31. (**i**) Untreated normal cornea; (**ii**) control eye treated with vehicle alone; (**iii**) 33DFTG-treated cornea. (**B**) The density of blood vessels covering the whole cornea was quantified by ImageJ. Blood vessels cover ∼40% and 28% of cornea in vehicle- and 33DFTG-treated mice, respectively. *n* = 13. Data from three independent experiments were plotted and analyzed with Student's *t*-test. *Scale bar*: 1 mm.

Galectin-3 has been shown to induce pathological angiogenesis in mouse models of corneal neovascularization.^27,28^ To determine whether inhibiting endogenous galectin-3 attenuates pathological angiogenesis, silver nitrate–cauterized mouse corneas were treated with local subconjunctival injections of 33DFTG (50 μM in 0.5% DMSO/PBS, *n* = 13) or vehicle alone (0.5% DMSO/PBS, *n* = 13) on days 0, 2, and 4 post surgery. Corneas were harvested on day 5 post cautery and stained with an anti-CD31 antibody to visualize blood vessels. As expected, vehicle-treated mouse eyes exhibited robust corneal angiogenesis (Fig. 1). The extent of corneal angiogenesis was significantly reduced in the inhibitor-treated eyes (∼30% reduction, *n* = 13 for each group) (Fig. 1).

### Galectin-3 Inhibition Attenuates VEGF-A–Induced Endothelial Cell Migration and Sprouting

**Figure 2:**
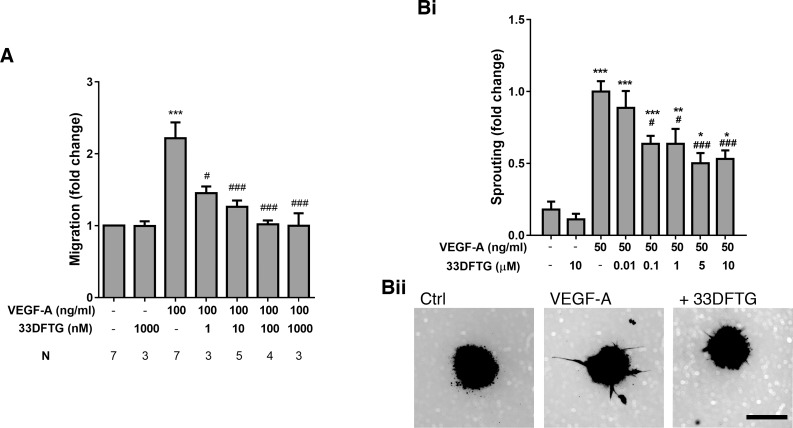
Galectin-3 inhibition by 33DFTG abolishes VEGF-A–induced endothelial cell migration and attenuates VEGF-A–induced endothelial cell sprouting. (**A**) HUVECs were serum starved overnight, detached with Accutase, resuspended in 1% FBS/M199, and added in the upper chamber. The bottom chamber was filled with VEGF-A in the presence or absence of varying concentrations of 33DFTG in 1% FBS/M199. After a 3-hour incubation, HUVECs that migrated to lower side of the membrane were counted. The inhibitor at as low as 1 nM concentration significantly reduced VEGF-A–induced chemotaxis. A value of 1.0 was assigned to vehicle-treated cells. The value of cell migration in response to VEGF-A and 33DFTG is expressed as fold change in migration with respect to the vehicle-treated cells. (**B**) HUVEC spheroids were prepared as described in Methods. Spheroids were seeded into type I collagen gels in the presence or absence of 33DFTG. After 6-hour pretreatment, spheroids were treated with VEGF-A (50 ng/mL) in the presence or absence of 33DFTG. After 24 hours, spheroids were stained with calcein AM and fluorescent images were acquired using the EVOS FL cell imaging system. Cumulative sprout lengths were quantified by ImageJ. (**i**) A value of 1.0 was assigned to the sprout length of VEGF-A–treated spheroids. The value of sprouting in response to vehicle alone or VEGF-A with varying doses of 33DFTG is expressed as fold change in sprouting with respect to the VEGF-A–treated spheroids. Each condition had 11 to 15 spheroids. (**ii**) Representative fluorescence images are shown. Data are plotted as mean ± SEM and analyzed with 1-way ANOVA. *P < 0.05 versus control; ***P < 0.001 versus control; ^###^*P* < 0.001 versus VEGF-A. (**B**) Representative images of sprouts are shown. Ctrl: 0.05% DMSO; VEGF-A: 50 ng/mL; +33DFTG: VEGF-A (50 ng/mL) + 33DFTG (5 μM). *Scale bar*: 100 μm.

Since VEGF-A/VEGFR-2 signaling pathway has been shown to be modulated by galectin-3,^27,28^ next we examined the effect of 33DFTG treatment on VEGF-A–induced angiogenesis in vitro. Two distinct in vitro angiogenesis assays, HUVEC cell migration and sprouting assays, were used. In the Boyden chamber–based cell migration assays, HUVECs were stimulated by placing VEGF-A (100 ng/mL) in the presence or absence of varying doses of 33DFTG (1–1000 nM) in the lower chambers. As expected, VEGF-A induced HUVEC cell migration (Fig. 2A). Importantly, 33DFTG inhibited VEGF-A–induced cell migration in a dose-dependent manner (Fig. 2A). At 100 nM concentration, 33DFTG completely abolished VEGF-A–induced cell migration.

The 3D sprouting assay bridges the gap between in vitro and in vivo assays, and provides an excellent approximation to in vivo angiogenesis. In the sprouting assay, HUVEC spheroids were embedded in the collagen matrix and stimulated with VEGF-A (50 ng/mL) in the presence or absence of varying doses of 33DFTG (from 0.01 to 10 μM). The presence of 33DFTG markedly inhibited VEGF-A–induced sprouting in a dose-dependent manner in the concentration range of 0.01 to 5 μM (Fig. 2B). Overall, the extent of inhibition of VEGF-A–induced HUVEC sprouting was ∼12% and ∼50% in the presence of 0.01 and 5 μM 33DFTG, respectively.

### The Galectin-3 Inhibitor Is Not Cytotoxic to Endothelial Cells

To assess the cytotoxic effect of 33DFTG, two reagents, calcein acetoxymethyl ester (calcein AM) and WST-1, were used. Calcein AM is a cell-permeant dye that is converted to a cell-impermeant green-fluorescent calcein and retained in cells with undamaged plasma membrane once it is hydrolyzed/deesterified by intracellular active esterases. In this assay, high calcein fluorescent intensity was detected in 33DFTG-treated cells, and there was no difference in the fluorescence intensity between 33DFTG-treated and vehicle-treated control cells (Supplementary Fig. S1A). In contrast, cells treated with 0.1% Triton X-100 exhibited minimal calcein fluorescence intensity (Supplementary Fig. S1A).

WST-1 is a tetrazolium salt that is converted to formazan by mitochondrial and plasma membrane dehydrogenases. Consistent with the results obtained by calcein AM viability assay, similar OD values were detected in untreated cells, cells treated with 33DFTG, and control cells treated with vehicle alone, whereas the OD values of cells treated with 0.1% Triton X-100 were at background levels (Supplementary Fig. S1B). Taken together, our results demonstrate that the antiangiogenic effect of 33DFTG is not due to cytotoxicity to the endothelial cells.

### Galectin-3 Inhibition Attenuates Corneal Fibrosis

**Figure 3:**
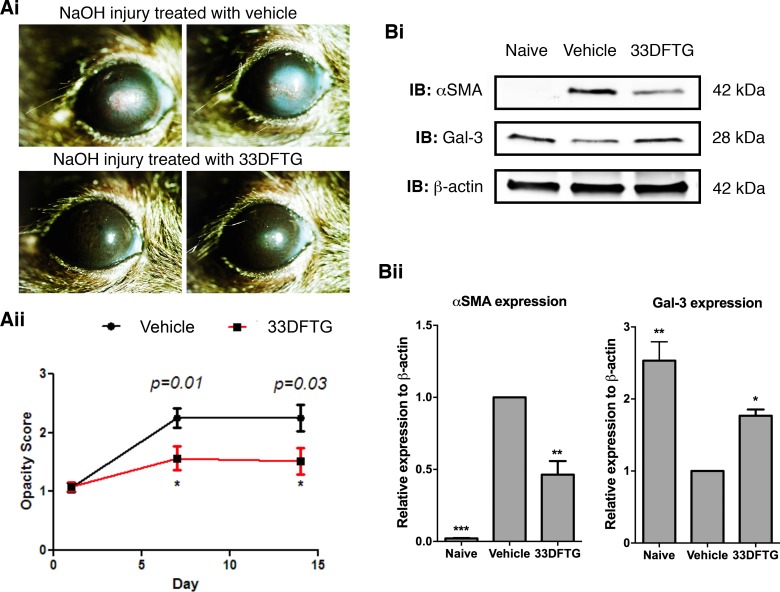
Galectin-3 inhibition by 33DFTG ameliorates corneal fibrosis. (**A**) Treatment with the inhibitor reduces corneal opacification. Mouse corneas were injured by alkali burn as described in Methods. One day post alkali burn, mice were equally divided into two groups. One group of mice was treated with 33DFTG (50 μM in 10 μL) by local subconjunctival injections on alternate days from day 1 until day 13. Control mice were injected with 10 μL PBS containing 0.5% DMSO (vehicle). Opacity score was recorded on days 1, 7, and 14. (**i**) Two representative photomicrographs of day 14 post injury of each group are shown. (**ii**) Opacity scores of three independent experiments are shown. *n* = 28 for vehicle (0.5% DMSO/PBS)-treated group; *n* = 29 for 33DFTG-treated group. (**B**) Expression of α-SMA is reduced in the corneas of 33DFTG-treated eyes. On day 14 post injury, corneal lysates (containing 30 μg protein) from injured eyes along with untreated normal eyes were subjected to electrophoresis in 4% to 15% SDS-PAGE gels. Protein blots of the gels were probed with anti-α-SMA, anti-galectin-3, and anti-β-actin antibodies. (**i**) Representative immunoblots are shown. (**ii**) Relative band intensity was quantified by Image Studio. Expression value of α-SMA and galectin-3 was normalized to β-actin, a value of 1.0 was given to vehicle (0.5% DMSO/PBS)-treated corneas, and the expression values of α-SMA and galectin-3 in the 33DFTG (50 μM)-treated corneas were calculated as fold changes. Four corneas were pooled and considered one biological replica. *n* = 3. Data are plotted as mean ± SEM and analyzed using 1-way ANOVA. *P < 0.05, **P < 0.01, ***P < 0.001 versus vehicle.

**Figure 4:**
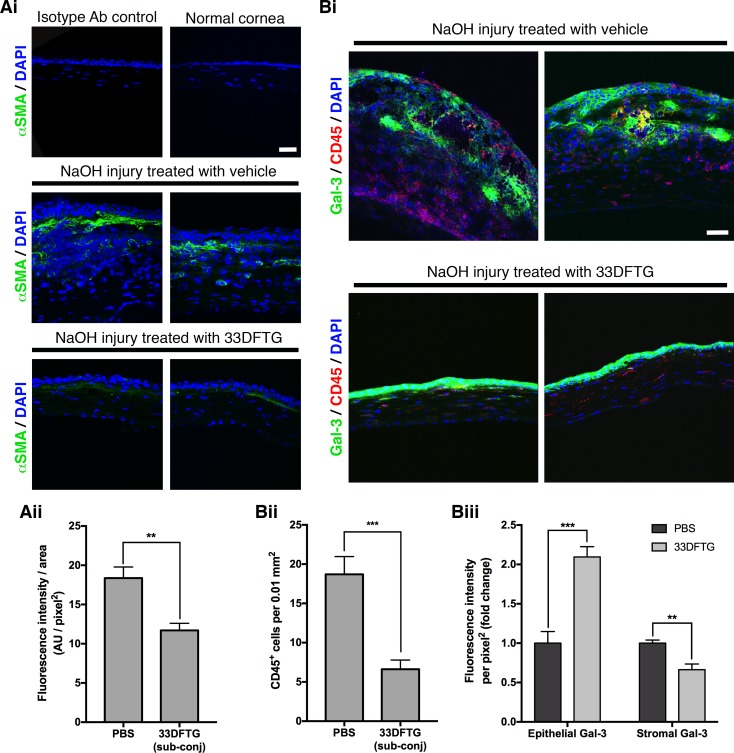
Treatment with the galectin-3 inhibitor decreases α-SMA immunoreactivity, inhibits CD45^+^ cell infiltration, and affects galectin-3 expression and distribution in alkaline-burned corneas. (**A**) Frozen tissue sections of vehicle (0.5% DMSO in PBS)- and 33DFTG (50 μM)-treated NaOH-cauterized eyes collected on day 14 post injury were immunostained with anti-α-SMA (*green*), followed by counterstaining with DAPI (*blue*). (**i**) No immunoreactivity was detected in corneas that were stained with isotype control IgG (*top left*). In normal control cornea, no α-SMA immunoreactivity was detected (*top right*). In contrast, α-SMA immunoreactivity was observed in subepithelial stroma in alkali-burned corneas (*middle*). Markedly reduced α-SMA immunoreactivity was detected in the 33DFTG-treated corneas (*bottom*). (**ii**) Fluorescence intensity (arbitrary unit, AU) of α-SMA within corneal stroma was quantified by ImageJ software. (**B**) Frozen tissue sections of vehicle- and 33DFTG-treated eyes collected on day 14 post injury were immunostained with anti-galectin-3 (*green*), anti-CD45 (*red*), and DAPI (*blue*). (**i**) Compared to vehicle-treated controls, 33DFTG-treated corneas exhibited reduced infiltration of CD45^+^ cells, increased galectin-3 immunoreactivity in corneal epithelium, and decreased galectin-3 immunoreactivity in corneal stroma. (**ii**) Cell numbers and fluorescence intensity were quantified. Immunostaining processing and fluorescence exposure time of all images are the same. Two representative images of vehicle- and 33DFTG-treated corneas are shown. For both (**A**) and (**B**), vehicle control, *n* = 4; 33DFTG, *n* = 6. Data are plotted as mean ± SEM and analyzed with Student's *t*-test. **P < 0.01; ***P < 0.001. *Scale bar*: 20 μm.

Since galectin-3 plays a key role in fibrosis in nonocular tissues,^29–32^ we sought to determine whether 33DFTG ameliorates corneal fibrosis in a mouse model of alkaline burn injury. To test this, mouse corneas were injured by alkali burn and treated with 33DFTG (50 μM in 10 μL) or vehicle (10 μL 0.5% DMSO in PBS) by subconjunctival injections on alternate days from day 1 until day 13. Corneal opacification was scored on days 1, 7, and 14 post injury. On both days 7 and 14 post injury, corneal opacity was markedly reduced in 33DFTG-treated eyes compared to the vehicle-treated eyes (Fig. 3A, *n* ≥ 28). To quantitate the extent of fibrosis in vehicle- and 33DFTG-treated corneas, tissue lysates of mouse corneas from both groups were collected on day 14 post surgery and were subjected to Western blot analyses to quantify α-SMA (a myofibroblast marker) expression levels. Fold-change values in α-SMA expression levels relative to β-actin expression are shown in Figure 3B. As expected, α-SMA expression level was minimal in normal untreated corneas (Fig. 3B). In contrast, consistent with the reduced corneal opacification detected in 33DFTG-treated eyes, the expression level of α-SMA was markedly reduced in 33DFTG-treated corneas (∼55% reduction, Fig. 3B). To investigate expression pattern of α-SMA at the tissue level, frozen sections of vehicle-treated control (0.5% DMSO/PBS) and 33DFTG-treated corneas were immunostained with anti-α-SMA antibody. Consistent with the Western blot result (Fig. 3B), immunoreactivity of α-SMA was markedly reduced in 33DFTG-treated corneas compared with the corneas treated with vehicle alone (∼36% reduction, Fig. 4), suggesting that 33DFTG treatment reduces myofibroblast activity and/or accumulation.

Our previous study demonstrated that galectin-3 expression is markedly reduced in silver nitrate−cauterized mouse corneas as compared with normal untreated mouse corneas,^22^ and that the reduced expression of galectin-3 was largely due to the downregulation of galectin-3 in the corneal epithelium.^22^ To determine whether galectin-3 expression in NaOH−cauterized mouse corneas is also reduced, tissue lysates of mouse corneas from both groups were collected on day 14 post surgery, and were subjected to Western blotting by using anti-galectin-3 antibody. Consistent with our previous results,^22^ galectin-3 expression level was markedly decreased in NaOH-cauterized corneas as compared with untreated normal corneas (∼60% reduction, Fig. 3B). Interestingly, in the current study, in NaOH−injured corneas treated with 33DFTG, galectin-3 expression level was higher as compared with vehicle-treated corneas (Fig. 3B). In addition, in line with our Western blot results showing that galectin-3 expression is markedly increased in the 33DFTG-treated corneas (Fig. 3B), immunohistochemical analyses revealed that galectin-3 immunoreactivity was increased in corneal epithelium of the 33DFTG-treated corneas (∼120% increase, Figs. 4Bi, 4Biii). However, in contrast to corneal epithelium, galectin-3 expression in corneal stroma of the inhibitor-treated eyes was reduced (∼34% reduction, Figs. 4Bi, 4Biii). The mechanism by which galectin-3 inhibition influences galectin-3 expression in a cell/tissue-specific manner is not known. In any case, our results suggest that galectin-3 inhibition dampens alkaline burn–induced inflammation and fibrosis.

Since galectins are immunomodulatory molecules, we sought to determine if galectin-3 inhibition decreases injury-induced inflammation in our mouse model of corneal fibrosis. For this purpose, frozen sections of vehicle- and 33DFTG-treated corneas on day 14 post injury were immunostained with an antibody against CD45 (a cellular marker for matured differentiated hematopoietic cells). As expected, NaOH-cauterized corneas were infiltrated with many CD45^+^ cells. Infiltration of CD45^+^ cells was greatly diminished in corneas treated with the galectin-3 inhibitor (∼65% reduction, Figs. 4Bi, 4Bii).

**Figure 5:**
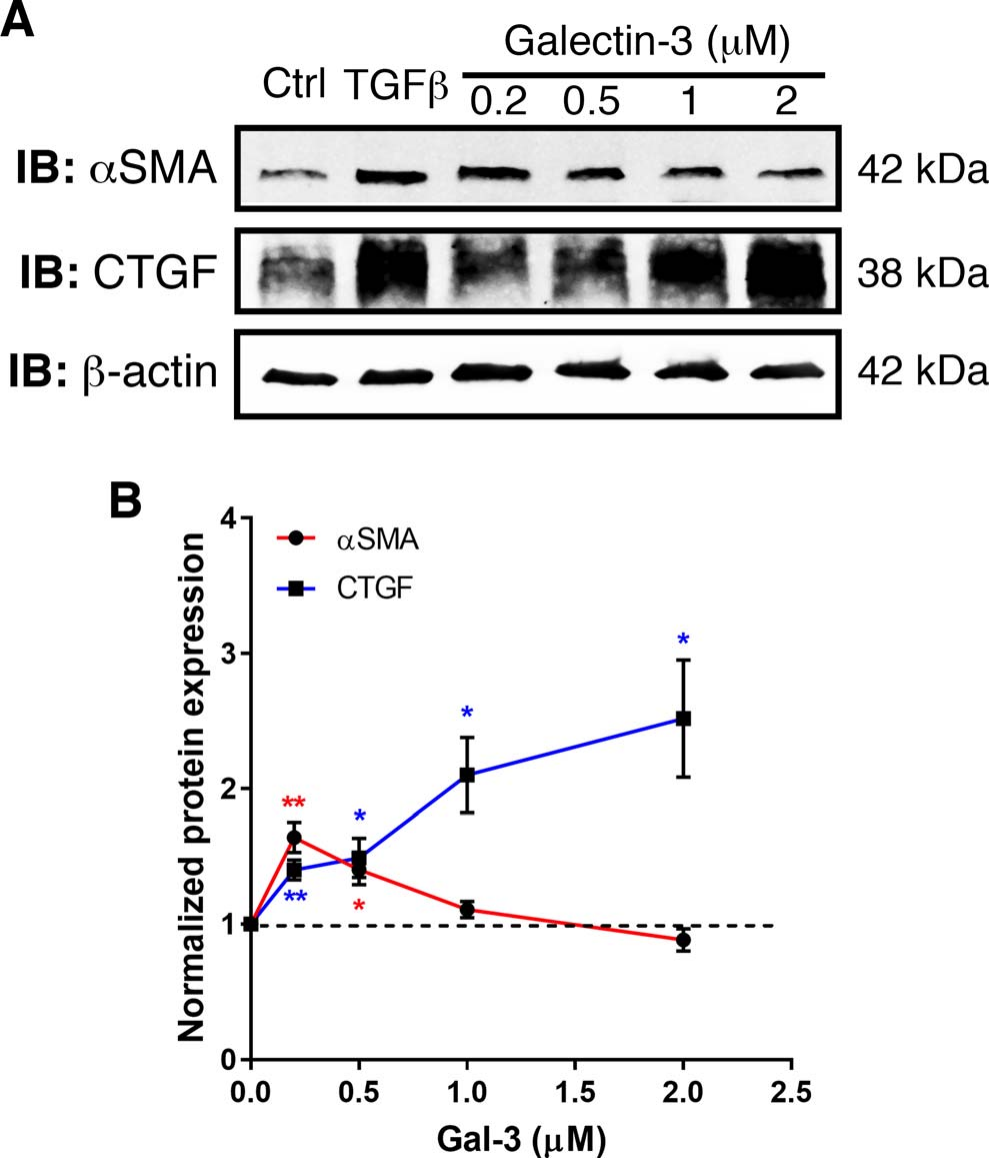
Galectin-3 treatment increases expression of fibrosis-related proteins. Primary human corneal fibrocytes were treated overnight with TGF-β1 (0.5 ng/mL in MEM, a positive control) and varying doses of recombinant galectin-3 ranging from 0.2 to 2 μM. Cell lysates containing 30 μg protein were subjected to electrophoresis in 4% to 15% SDS-PAGE gels. Protein blots of the gels were probed using anti-α-SMA, anti-CTGF, and anti-β-actin as described in Methods. (**A**) Representative immunoblots from three independent experiments with the same conclusion are shown. (**B**) Relative band intensity quantified by Image Studio. Expression value of α-SMA (*red line*) and CTGF (*blue line*) was normalized to β-actin. A value of 1.0 was assigned to the untreated control cells, and values of cells treated with galectin-3 are expressed as fold change with respect to the untreated control cells. Data from three independent experiments are plotted as mean ± SEM and analyzed using Student's *t*-test. *P < 0.05, **P < 0.01 versus untreated control cells.

The inhibitor 33DFTG blocks the carbohydrate recognition of galectin-3 to efficiently neutralize the carbohydrate-mediated function of galectin-3 outside of the cells. To determine whether galectin-3 is expressed on cell surface of corneal fibroblasts, we employed flow cytometry analysis to assess the cell surface expression of galectin-3 in the primary human corneal fibroblasts. In this experiment, human corneal epithelial cells were included as a positive control. Consistent with a previous study in our laboratory,^42^ galectin-3 was expressed on the corneal epithelial cell surface (Supplementary Fig. S2). In contrast, galectin-3 was not detectable on the cell surface of the human corneal fibroblasts (Supplementary Fig. S2). However, there was abundant expression of intracellular galectin-3 in the fibroblasts. The molecular mechanism by which galectin-3 differentially distributes in each cell type has not been fully investigated and is beyond the scope of the current study. In any case, since paracrine functions of galectins are well known and we have shown that galectin-3 is expressed abundantly in ECM of injured corneas,^22,23^ we hypothesize that secreted galectin-3 from the injured corneal epithelium and infiltrated cells may activate corneal fibrocytes to differentiate into myofibroblasts. To test this hypothesis, the primary human corneal fibroblasts were treated with recombinant galectin-3 or TGF-β1 (a positive control) and the cell lysates were subjected to Western blotting using anti-α-SMA and anti-CTGF (a fibrosis-related growth factor). As expected, TGF-β1 markedly upregulated expression of α-SMA and CTGF in the corneal fibroblasts (Fig. 5). Similarly, recombinant galectin-3 increased expression of α-SMA and CTGF in these cells (Fig. 5). Notably, galectin-3 treatment induced the expression of CTGF in a dose-dependent manner, whereas α-SMA expression induced by galectin-3 treatment was bell-shaped: increased at 0.2 μM, but similar to or decreased at 2 μM compared with untreated cells (Fig. 5). Future investigations are needed to address the biological relevance of this observation.

### The Eye Drop Formulation of the Inhibitor Reduces Corneal Angiogenesis

**Figure 6:**
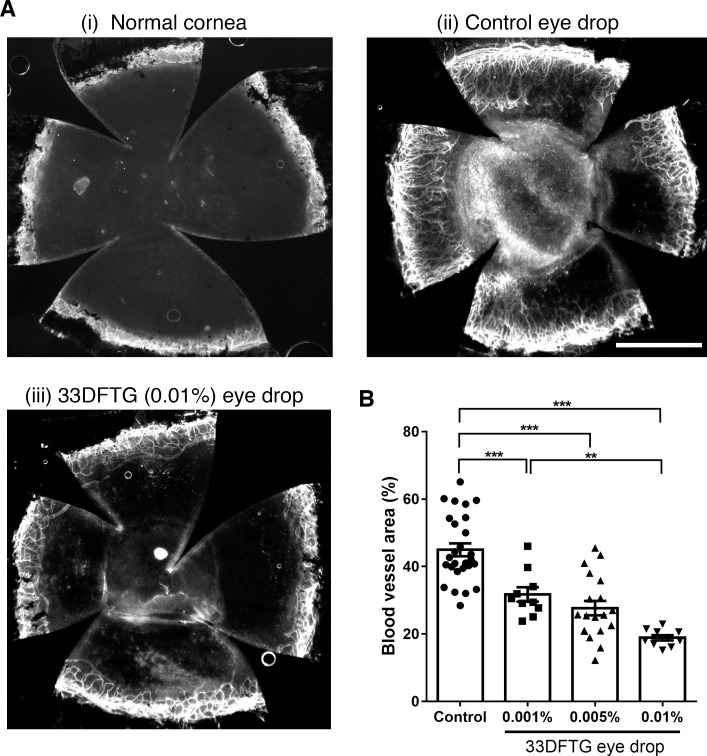
Eye drop formulation of 33DFTG reduces cautery-induced corneal angiogenesis. Corneal neovascularization was induced by silver nitrate cautery as described in Methods. Ten microliters of varying doses of 33DFTG in 1.65% HEC or vehicle (1.65% HEC) alone was applied twice daily. After 7 days, mice were killed, and flat mounts of corneas were stained with anti-CD31 to visualize blood vessels. (**A**) Representative corneal flat mounts stained with anti-CD31. (**i**) Untreated normal cornea; (**ii**) vehicle control eye drop–treated cornea; (**iii**) 33DFTG (0.01%) eye drop–treated cornea. (**B**) The density of blood vessels covering the whole cornea was quantified by ImageJ. Blood vessels cover 45% and 19% of cornea in vehicle- and 33DFTG-treated mice, respectively. *n* ≥ 10 per group. Data are plotted and analyzed with 1-way ANOVA. **P < 0.01, ***P < 0.001. *Scale bar*: 1 mm.

Topical eye drop is the most convenient route of drug administration, especially for the treatment of anterior segment diseases. However, precorneal factors (blinking, tear film, tear turnover, and so on) and rapid solution drainage (15–30 seconds after instillation in humans) profoundly impact the bioavailability of topical agents.^43^ To overcome these hurdles, we have developed an eye drop formulation (1.65% HEC) for 33DFTG. This eye drop formulation does not use DMSO to dissolve 33DFTG; therefore, by using this eye drop formulation we can eliminate any potential toxicity effect caused by DMSO. To determine the antiangiogenic efficacy of the 33DFTG eye drops, mouse corneas were injured by silver nitrate cautery and 33DFTG eye drops were instilled twice a day for 7 days. As a control, the eye drop formulation (1.65% HEC) without the loading of 33DFTG was employed. On day 7 post injury, mouse corneas were harvested and stained with an anti-CD31 antibody to visualize blood vessels. In the corneal whole-mount staining, blood vessel areas in the mouse corneas treated with vehicle eye drop formulation were similar to the blood vessel areas in the corneas treated with subconjunctival injections of PBS (Figs. 1, 6), indicating that HEC alone has no significant effect on corneal angiogenesis. In sharp contrast, 33DFTG-loaded eye drops markedly decreased silver nitrate cautery–induced corneal angiogenesis in a dose-dependent manner (Fig. 6). At the maximum concentration (0.01%, which is equal to 154.2 μM), 33DFTG eye drops reduced corneal angiogenesis by ∼60% as compared to control eye drops (mean blood vessel area: 33DFTG at 0.01% treatment, 18.9%; vehicle treatment, 45.0%; Fig. 6). Taken together, these findings demonstrated that 33DFTG eye drop formulation can efficiently curtail corneal angiogenesis.

## Discussion

Recent studies have demonstrated that galectin-3 promotes angiogenesis^27,28^ as well as fibrosis^30^ and that it does so by independent mechanisms. Thus, the current study was designed to assess the potential of targeting galectin-3 to develop a dual-benefit drug to control both neovascularization and fibrosis of ocular tissues using cornea as a model. We demonstrated here that a small-molecule inhibitor targeting galectin-3, 33DFTG, attenuates (1) corneal neovascularization as evidenced by reduction in CD31^+^ blood vessels in silver nitrate–cauterized mouse corneas, and (2) fibrosis and myofibroblast differentiation as evidenced by reduced corneal opacity and α-SMA expression in alkali-injured mouse corneas. We further show that 33DFTG also blocks VEGF-A–mediated HUVEC migration and sprouting. The extent of reduction in corneal neovascularization in vivo and HUVEC migration and sprouting in vitro by 33DFTG was similar to that we previously reported in galectin-3 null mice and galectin-3 knockdown HUVECs.^27,28^ These results further confirm that galectin-3 is the major galectin that promotes pathological angiogenesis in the cornea. In addition, since 33DFTG blocks the carbohydrate-binding site of galectin-3,^31^ the antiangiogenic effect of 33DFTG observed in this study further supports the concept that the carbohydrate recognition by galectin-3 is essential for its proangiogenic function. Indeed, previous results in our laboratory demonstrated that knockdown of GnTV, an enzyme that synthesizes high-affinity glycan ligands for galectin-3, inhibits VEGF-induced HUVEC migration and tube formation, and that VEGF-induced corneal angiogenesis is attenuated in GnTV null mice.^28^ Taken together, our findings suggest that 33DFTG inhibits corneal angiogenesis by blocking the interaction between the carbohydrate recognition domain of galectin-3 and the GnTV-synthesized N-glycans.

Thus far, the profibrotic role of galectin-3 has not been investigated in ocular tissues. Our findings that 33DFTG treatment ameliorates chemical injury–induced mouse corneal fibrosis are consistent with reports demonstrating profibrotic role of galectin-3 in nonocular tissues.^29–32^ Myofibroblast accumulation has been identified as the critical factor that leads to corneal fibrosis.^44–46^ The present findings that 33DFTG treatment reduces expression (Fig. 3) and distribution (Fig. 4) of α-SMA (a widely accepted marker for myofibroblasts) in NaOH-burned mouse corneas suggest that inhibiting galectin-3 by 33DFTG attenuates corneal fibrosis, in part, through curtailing differentiation of stromal cells to myofibroblasts in the injured mouse cornea. The antifibrotic effect of 33DFTG on corneal fibrosis may also be due to its suppressive effect on epithelial-to-mesenchymal transition (EMT), a process that is thought to be involved in the development of subepithelial corneal fibrosis.^47^ In support of this concept, it has been reported that in lung epithelial cells, galectin-3 deficiency results in reduced retention of TGF-βRII at the cell surface and decreased β-catenin activation,^31^ and thereby prohibits TGF-β–induced EMT. Alternatively, based on our previous findings that galectin-3 is highly upregulated in inflamed mouse corneal stroma,^22,23^ and the findings in this study that galectin-3 induces expression of fibrosis-related proteins (α-SMA and CTGF, Fig. 5), it is possible that 33DFTG treatment attenuates corneal fibrosis by neutralizing the effect of secreted galectin-3 in the corneal stroma, which may be derived from inflammatory cells and injured epithelial cells.^48^ Future studies involving the use of cell type–specific knockout of galectin-3 will delineate the contribution of various cell types in the regulation of corneal fibrosis. Our result that galectin-3 induces CTGF expression in corneal stromal cells suggests that galectin-3 may exacerbate corneal fibrosis/scarring partially through upregulation of CTGF. In this respect, it is known that CTGF is overexpressed in several fibrotic disorders (reviewed in Ref. 49). In the cornea, CTGF is upregulated in both fibroblasts and epithelium after corneal injuries.^50,51^ Moreover, an in vitro study using corneal fibroblasts has demonstrated that CTGF is required for TGF-β–mediated induction of collagen synthesis.^50^ In fact, evidence from nonocular studies using animal models suggests that TGF-β with excess CTGF may result in chronic fibrosis (reviewed in Ref. 52). The interplay among TGF-β, CTGF, and galectin-3 has not been explored; however, considering that galectin-3 is required for TGF-β–mediated signal transduction,^31^ that galectin-3 acts upstream of CTGF (Fig. 5), and that galectin-3 null mice are overall healthy, targeting galectin-3 instead of TGF-β or CTGF has the potential to effectively block pathological fibrosis with minimal adverse effects.

As mentioned above, bevacizumab treatment is efficient in only ∼55% of patients with the wet form of age-related macular degeneration.^12,13^ The limited success of the VEGF targeting therapy is due partially to maturation of established vessels. A hallmark of vessel maturation and stability is pericyte coverage of vasculature (reviewed in Ref. 53). According to published studies, targeting galectin-3 may interfere with pericyte differentiation/maturation and recruitment by two possible mechanisms. First, targeting galectin-3 may interrupt TGF-β−mediated pericyte maturation during the process of angiogenesis. This notion is supported by previous studies showing that TGF-β−mediated signaling pathway plays a key role in modulating pericyte differentiation,^53^ and that galectin-3 modulates TGF-β signaling pathway.^31^ Second, inhibiting galectin-3 may reduce pericyte recruitment by abolishing the interaction between galectin-3 and NG2 proteoglycan, a cellular marker for pericytes of angiogenic vessels.^54^ This concept is supported by published studies demonstrating that (1) galectin-3 binds to NG2 proteoglycan,^55^ (2) NG2 proteoglycan−promoted cell migration is dependent on galectin-3,^55^ and (3) pericyte migration is a key event during blood vessel maturation.^53^ Thus it seems plausible that galectin-3 inhibition may interfere with pericyte recruitment and thereby mute the maturation of blood vessels. In such a case, targeting galectin-3 may be an attractive option for anti-VEGF nonresponders.

Although 33DFTG has at least 10-fold greater selectivity for galectin-3 over a number of other mammalian galectins that are expressed in the cornea (including galectins-7, -8, and -9),^22,23^ it does bind to galectin-1 with similar affinity (33DFTG affinity for mammalian galectins: galectin-1, 12 nM; galectin-3, 14 nM; galectin-7, 1900 nM; galectin-8N, 86000 nM; galectin-9N, 680 nM; galectin-9C, 120 nM).^38^ In a recent study, we have demonstrated that in normal mouse corneas, galectin-1 can be detected by real-time PCR and Western blotting,^22,23^ and that galectin-1 expression is upregulated in the inflamed mouse corneas.^22,23^ Therefore, we cannot exclude the possibility that in the current study, 33DFTG may have also targeted galectin-1 in addition to galectin-3. Although the role of galectin-1 in ocular angiogenesis has not been established, accumulating evidence has demonstrated that galectin-1 plays a pivotal role in modulating tumor angiogenesis. Tumors that resist the anti-VEGF therapy secrete high amounts of galectin-1, which promotes angiogenesis by activation of VEGFR-2 in a ligand (i.e., VEGF)-independent manner.^56^ In contrast, blocking galectin-1 with a neutralizing antibody markedly attenuates tumor angiogenesis.^57^ Considering that both galectins-1 and -3 regulate tumor angiogenesis,^56–59^ dual targeting galectins-1 and -3 by 33DFTG may be a novel therapeutic approach for tumor angiogenesis.

The role of galectin-1 in the pathogenesis of fibrosis has been controversial. One study has suggested that galectin-1 may promote the TGF-β–induced pulmonary fibrosis.^60^ In contrast, another study has suggested that galectin-1 suppresses TGF-β–induced renal fibrosis under high-glucose conditions.^61^ Clearly, more direct studies involving the use of galectin-1 knockout mice are needed to elucidate the role of galectin-1 in fibrosis in vivo. Regardless of the mechanism, the positive therapeutic effect of the small-molecule inhibitor 33DFTG in two different mouse models of corneal injuries suggests that galectin-3 has the potential to be a promising target for treatment of myriad ocular diseases characterized by excessive angiogenesis and fibrosis. In addition, 33DFTG eye drop formulation developed in this study provides a convenient route of drug administration and can be useful for clinical purposes in the future.

## Supplementary Material

Supplement 1
