# Supplementary material for: Galectin-3 Inhibition by a Small-Molecule Inhibitor Reduces Both Pathological Corneal Neovascularization and Fibrosis
Source: Invest Ophthalmol Vis Sci. 2017 Jan;58(1):9–20. doi: 10.1167/iovs.16-20009 (PMC5225999; doi:10.1167/iovs.16-20009)
Supplement: Supplement 1 [file iovs-57-15-05_s01.pdf]

Supplementary Figure 1

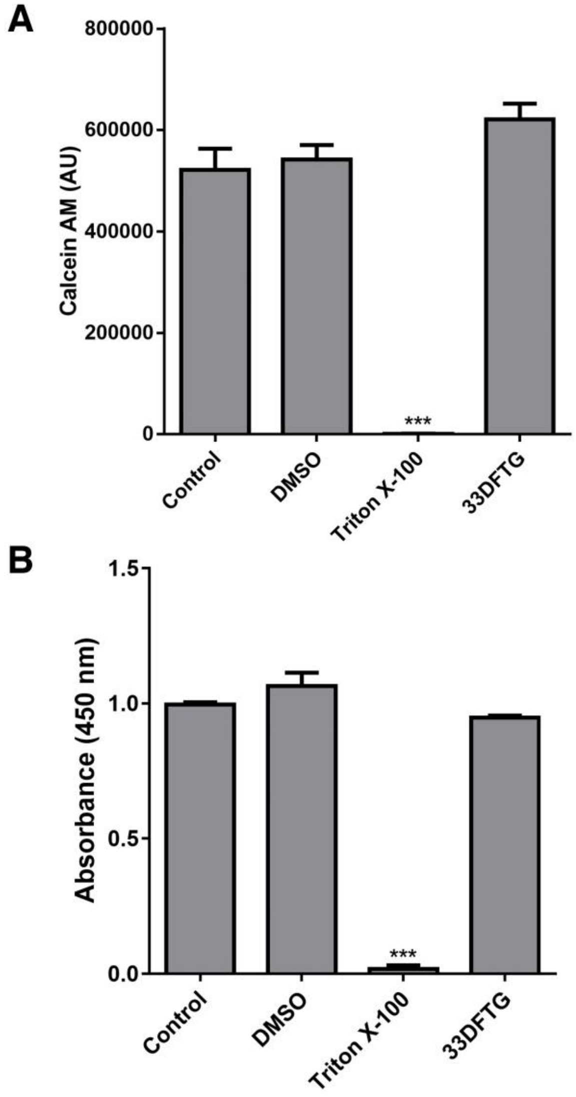

Supplementary Figure 1. The galectin-3 inhibitor is nontoxic to HUVECs. HUVECs were incubated in 1% FBS/M199 overnight and treated with 0.05% DMSO, 0.1% Triton X-100, and 5 μM 33DFTG overnight. (A) Calcein AM was incubated with the cells for 30 min. (B) WST-1, a tetrazolium salt, was incubated with the cells for 2 hr. Signals were detected by a spectrophotometer. HUVEC viability is not reduced after 33DFTG or 0.05% DMSO treatment. As expected, no viable cells were detected after Triton X-100 treatment. Data are plotted as mean±SEM and analyzed with one-way ANOVA. ***P<0.001 vs control.

Supplementary Figure 2

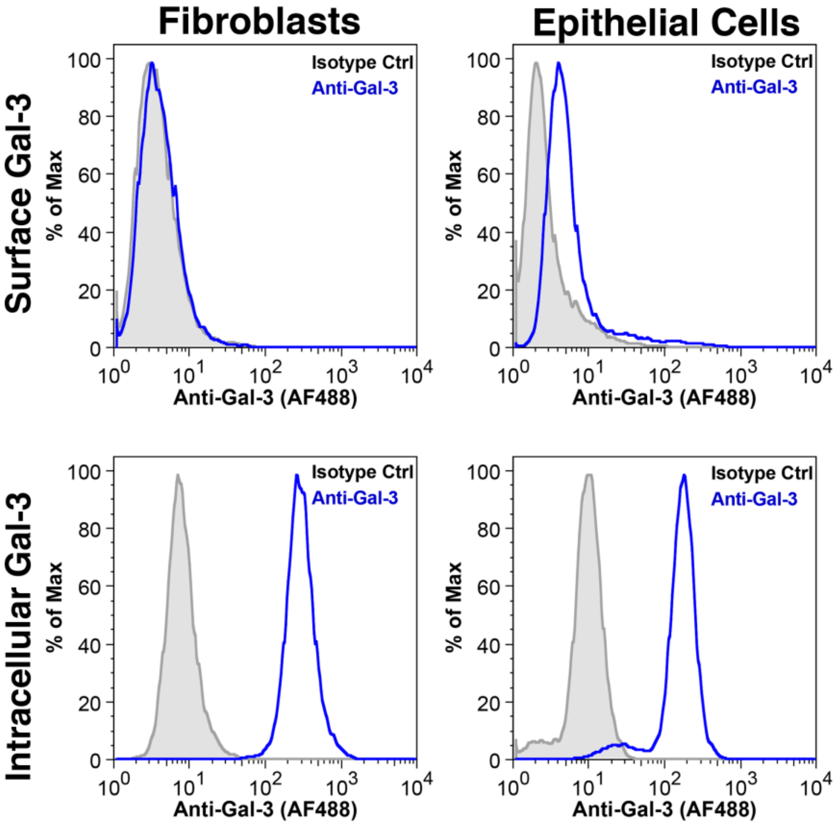

Supplementary Figure 2. Galectin-3 is not expressed on cell surface of corneal stromal cells. Flow cytometry analysis was used to assess the intracellular (lower panel) and cell surface (upper panel) expression of galectin-3 in corneal fibroblasts and epithelial cells. Grey lines: cells stained with isotype control antibody; blue lines: cells stained with anti-galectin-3 antibody conjugated with Alexa Fluor 488. Cell surface galectin-3 was detected in corneal epithelial cells but not in corneal fibrocytes, whereas intracellular galectin-3 was detected in both cell types. Representative results from two independent experiments with the same conclusion are shown.
